# Statin treatment increases the clinical risk of tendinopathy through matrix metalloproteinase release – a cohort study design combined with an experimental study

**DOI:** 10.1038/s41598-019-53238-7

**Published:** 2019-11-29

**Authors:** Pernilla Eliasson, Franciele Dietrich-Zagonel, Anna-Carin Lundin, Per Aspenberg, Alicja Wolk, Karl Michaëlsson

**Affiliations:** 1 Department of Clinical and Experimental Medicine, Faculty of Health Sciences, Linköping University, Linköping, Sweden; 2 National Institute of Environmental Medicine, Division of Nutritional Epidemiology, Karolinska Institute, Stockholm, Sweden; 3 Section of Orthopedics, Department of Surgical Sciences, Uppsala University, Uppsala, Sweden

**Keywords:** Epidemiology, Mechanisms of disease, Risk factors

## Abstract

Recent experimental evidence indicates potential adverse effects of statin treatment on tendons but previous clinical studies are few and inconclusive. The aims of our study were, first, to determine whether statin use in a cohort design is associated with tendinopathy disorders, and second, to experimentally understand the pathogenesis of statin induced tendinopathy. We studied association between statin use and different tendon injuries in two population-based Swedish cohorts by time-dependent Cox regression analysis. Additionally, we tested simvastatin in a 3D cell culture model with human tenocytes. Compared with never-users, current users of statins had a higher incidence of trigger finger with adjusted hazard ratios (aHRs) of 1.50 for men (95% confidence interval [CI] 1.21–1.85) and 1.21 (1.02–1.43) for women. We also found a higher incidence of shoulder tendinopathy in both men (aHR 1.43; 1.24–1.65) and women (aHR 1.41; 0.97–2.05). Former users did not confer a higher risk of tendinopathies. *In vitro* experiments revealed an increased release of matrix metalloproteinase (MMP)-1 and MMP-13 and a weaker, disrupted matrix after simvastatin exposure. Current statin use seems to increase the risk of trigger finger and shoulder tendinopathy, possibly through increased MMP release, and subsequently, a weakened tendon matrix which will be more prone to injuries.

## Introduction

Statins are commonly used to lower blood cholesterol levels, which helps prevent cardiovascular disease^1–3^. Although they are generally believed to be safe, muscle toxicity is not uncommon^3,4^. Increasing attention has also been directed towards potential harmful effects on tendons, leading to tendinopathy or rupture. Case reports and pharmacovigilance data suggest an increased risk of tendon rupture and tendinopathy in statin users^4–8^, especially in men^7,9^; however, prospective cohort studies on the adverse effects of statin therapy are scarce and inconclusive^3,10–14^. The initial pharmacovigilance study describing a relationship between statins and tendon impairment reported “tendinitis” as the most frequent tendon adverse event with the highest incidence during the first year of statin use^7^. Simvastatin, is the most prescribed statin in Sweden and has together with atorvastatin and lovastatin the highest risk for muscle related side-effects, due to nonselective abilities to diffuse into other tissues than the liver^3^.

Tendinopathy, also known as tendinosis or tendinitis, is a broad term used to describe tendon degeneration^15^. The condition is characterized by collagen degeneration, fiber disorganization and increased amounts of ground substance. We have previously shown that trigger finger tendons show a histological appearance and gene expression pattern similar to tendinopathy at other locations, suggesting that this condition might be the most common form of tendinopathy. Because it is clinically an easily defined tendinopathy, it can serve as a suitable outcome variable in an observational study^16,17^.

An exact molecular mechanism of action for statins in tendon cells has not been established. *In vitro* studies have shown that extracellular matrix strength is reduced after statin treatment but, surprisingly, without altering the total levels of collagen^18^. This finding indicates that alterations in the balance of matrix metalloproteinases (MMPs) might play a role.

Given the indeterminate evidence on statin use and the risk of tendon pathology, we used two large Swedish population-based cohorts to evaluate a potential association between statin use and the risk of tendinopathy. We chose to study whether statins are associated with a higher risk of trigger finger and with tendinopathy in the shoulder or the Achilles tendon. Moreover, we used an *in vitro* model with artificial tendons (made from human tendon fibroblasts) to study the possible role of statin-driven MMP release in association with a weakened extracellular matrix.

## Results

To study the involvement of statin use in the development of tendon disorders a cohort study was performed with a time-dependent Cox regression analysis using two Swedish population-based cohorts in combination with three national registers. The baseline characteristics of the study participants (n = 92 933) from the Swedish Mammography Cohort (SMC) and the Cohort of Swedish Men (COSM) are shown in Table 1. The mean age at baseline was slightly <70 years. Statin use was common in both women (37%, n = 19 323) and men (44%, n = 17 854). The most frequent statin prescribed was simvastatin (69%), followed by atorvastatin (24%), rosuvastatin (4%) and pravastatin (2%).

**Table 1:** Descriptive characteristics of statin users and never-users.

	SMC (n = 52 220 women)		COSM (n = 40 713 men)	
	Statin users	Never-users	Statin users	Never-users
Number of women/men	19 323	32 987	17 854	22 859
Age, mean (SD)	68.4 (7.8)	69.5 (9.8)	69.5 (8.5)	66.6 (9.7)
Body mass index, mean (SD)	25.6 (4.0)	24.7 (3.9)	26.3 (3.3)	25.4 (3.1)
Metabolic equivalents (METs, in 1998)	42.4 (4.8)	42.5 (4.7)	41.5 (4.5)	41.7 (4.9)
Charlson weighted comorbidity index, mean (SD)	0.29 (0.72)	0.23 (0.64)	0.29 (0.65)	0.12 (0.43)
Energy intake (kcal/day)	1715 (577)	1731 (582)	2621 (820)	2714 (832)
Pack-years of smoking, mean (SD)	11.5 (10.5)	10.6 (10.1)	13.4 (15.9)	10.5 (14.2)
**Education, n (%)**				
≤9 years	12 751 (70.0)	21 944 (66.5)	10 078 (72.0)	16 603 (65.5)
10–12 years	3175 (16.4)	5412 (16.4)	1815 (13.0)	3910 (15.4)
>12 years	2585 (13.4)	5458 (16.6)	2045 (14.6)	4735 (18.7)
Vocational	38 (0.2)	82 (0.2)	57 (0.4)	84 (0.3)
Corticosteroid use, n (%)	1010 (5.2)	1540 (4.7)	3538 (19.8)	3710 (16.2)
Quinolone use, n (%)	2962 (15.3)	4438 (13.5)	6062 (34.0)	6516 (28.5)
Diabetes mellitus, n (%)	2618 (13.6)	1398 (4.2)	3963 (22.2)	1332 (5.8)
Renal insufficiency, n (%)	455 (2.4)	371 (1.1)	1002 (5.6)	547 (2.4)
Statin use, n (%)*				
Simvastatin	13 266 (68.7)	NA	12 256 (68.7)	NA
Pravastatin	415 (2.2)	NA	243 (1.4)	NA
Fluvastatin	43 (0.22)	NA	26 (0.2)	NA
Atorvastatin	4325 (22.4)	NA	4584 (25.7)	NA
Rosuvastatin	775 (4.0)	NA	745 (4.2)	NA

^*^% in statin user.

### Statin treatment is associated with an increased risk of trigger finger

**Figure 1:**
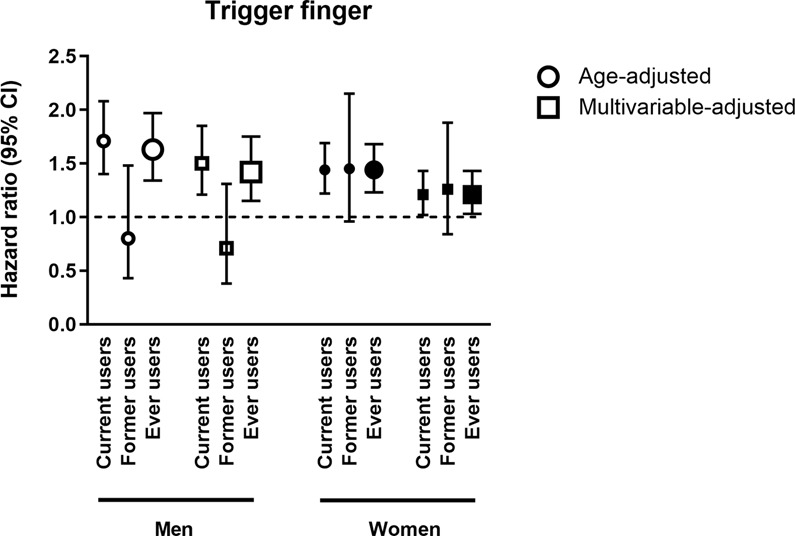
Adjusted hazard ratios (HRs) and 95% confidence intervals (CIs) for trigger finger. Adjusted hazard ratios (HRs) and 95% confidence intervals (CIs) for trigger finger in current, former and ever users of statins compared with never users (hazard ration 1.0). Circles indicate age-adjusted and squares multivariable-adjusted results. The multivariable-adjusted analyses were adjusted for age, BMI, METs, Charlson index, pack-years of smoking, energy intake, oral corticosteroid use, quinolone antibiotic use, diabetes mellitus, renal insufficiency and education level. The results are presented in white figures for men and black figures for women.

During 833 390 person-years of follow-up, we identified 1056 incident cases of trigger finger according to our ICD code criteria (626 women and 430 men). The incidence rates for trigger finger were 1.4/1000 person-years in women and 1.5/1000 in men. Compared with non-users, current users of statins conferred an overall higher risk of trigger finger: multivariable-adjusted hazard ratio (aHR) 1.50 (95% CI 1.21–1.85) for men and 1.21 (95% CI 1.02–1.43) for women (Fig. 1 and Table 2). The higher risk did not remain in former users. We found no relation between the duration of current statin use and the risk to develop trigger finger, i.e., the aHRs were similar in patients with 0–1 years versus >3 years of statin use. The highest apparent relative risk was seen in male users of rosuvastatin (aHR 2.19; 95% CI: 1.33–3.62) though this estimate is based on few cases. We found no statistically significant heterogeneity between the estimates for the different statins (p = 0.44 in women and p = 0.36 in men) or between prescribed doses.

**Table 2:** Hazard ratios (HR) and 95% confidence intervals (CIs) of trigger finger associated with statin use in women and men.

Statin use		Age-adjusted HR (95% CI)	Multivariable-adjusted^a^ HR (95% CI)	Age-adjusted HR (95% CI)	Multivariable-adjusted^a^ HR (95% CI)
		Women		Men	
Never use		1.0 (reference)	1.0 (reference)	1.0 (reference)	1.0 (reference)
Ever use	Any	1.44 (1.23–1.68)	1.21 (1.03–1.43)	1.63 (1.34–1.97)	1.42 (1.15–1.75)
	Low dose	1.35 (1.02–1.79)	1.15 (0.86–1.52)	0.97 (0.66–1.42)	0.92 (0.63–1.34)
	Medium dose	1.47 (1.24–1.74)	1.24 (1.04–1.47)	1.53 (1.25–1.86)	1.35 (1.09–1.66)
	High dose	1.14 (0.36–3.54)	0.92 (0.29–2.87)	1.04 (0.33–3.25)	0.98 (0.31–3.05)
	Simvastatin	1.30 (1.09–1.57)	1.12 (0.93–1.35)	1.50 (1.20–1.87)	1.33 (1.06–1.68)
	Pravastatin	1.83 (0.91–3.69)	1.63 (0.81–3.28)	1.48 (0.47–4.62)	1.28 (0.41–4.01)
	Fluvastatin	2.55 (0.36–18.12)	1.96 (0.27–14.04)	NA	NA
	Atorvastatin	1.61 (1.28–2.02)	1.33 (1.05–1.67)	1.76 (1.32–2.36)	1.51 (1.11–2.04)
	Rosuvastatin	1.89 (1.19–2.99)	1.50 (0.94–2.39)	2.62 (1.60–4.29)	2.19 (1.33–3.62)
Current use	Any	1.44 (1.22–1.69)	1.21 (1.02–1.43)	1.71 (1.40–2.08)	1.50 (1.21–1.85)
	0–1 years	1.37 (0.97–1.95)	1.16 (0.81–1.65)	1.66 (1.13–2.42)	1.48 (1.01–2.18)
	1–2 years	1.59 (1.08–2.36)	1.34 (0.90–1.99)	1.88 (1.23–2.87)	1.69 (1.10–2.59)
	2–3 years	2.13 (1.47–3.08)	1.76 (1.21–2.55)	1.78 (1.18–2.69)	1.58 (1.03–2.40)
	>3 years	1.41 (1.11–1.79)	1.12 (0.88–1.43)	1.66 (1.30–2.12)	1.43 (1.11–1.85)
Former use	Any	1.45 (0.96–2.15)	1.26 (0.84–1.88)	0.80 (0.43–1.48)	0.71 (0.38–1.31)
	<1 year ago	1.05 (0.50–2.21)	0.96 (0.45–2.02)	0.49 (0.16–1.54)	0.44 (0.14–1.38)
	1–2 years ago	1.38 (0.65–2.92)	1.28 (0.60–2.70)	0.47 (0.12–1.88)	0.43 (0.11–1.72)
	>2 years ago	1.33 (0.76–2.33)	1.24 (0.71–2.17)	0.82 (0.36–1.84)	0.76 (0.34–1.73)

^a^Multivariable-adjusted model adjusted for age, BMI, METs, Charlson index, pack-years of smoking, energy intake, oral corticosteroid use, quinolone antibiotic use, diabetes mellitus, renal insufficiency and education level.

### Ongoing statin use is also associated with an increased risk of shoulder tendinopathy

**Figure 2:**
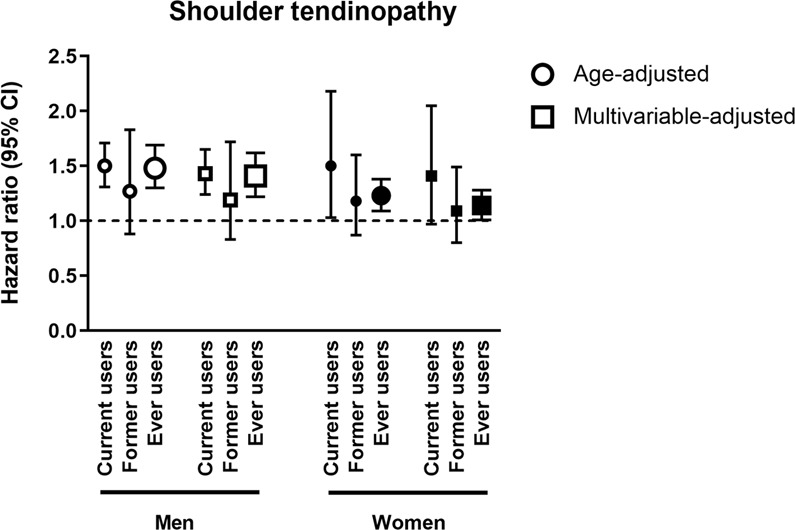
Adjusted hazard ratios (HRs) and 95% confidence intervals (CIs) for shoulder tendinopathy. Adjusted hazard ratios (HRs) and 95% confidence intervals (CIs) for shoulder tendinopathy in current, former and ever users of statins compared with never users (hazard ration 1.0). Circles indicate age-adjusted and squares multivariable-adjusted results. The multivariable-adjusted analyses were adjusted for age, BMI, METs, Charlson index, pack-years of smoking, energy intake, oral corticosteroid use, quinolone antibiotic use, diabetes mellitus, renal insufficiency and education level. The results are presented in white figures for men and black figures for women.

In the same cohorts we identified 1102 women and 957 men with shoulder tendinopathy (Fig. 2 and Table 3). We observed a higher incidence of shoulder tendinopathy in men and women who were current users of statins (aHRs 1.43; 95% CI: 1.24–1.65 and 1.41; 95% CI 0.97–2.05, respectively) compared with men and women who were never-users. Former users of statins were not affected by a higher incidence of shoulder tendinopathy. Again, we found no statistically significant heterogeneity between the estimates for the different statins (p = 0.78 in women and p = 0.10 in men).

**Table 3:** Hazard ratios (HR) and 95% confidence intervals (CIs) for shoulder tendinopathy associated with statin use in women and men.

Statin use		Age-adjusted HR (95% CI)	Multivariable-adjusted^a^ HR (95% CI)	Age-adjusted HR (95% CI)	Multivariable-adjusted^a^ HR (95% CI)
		Women		Men	
Never use		1.0 (reference)	1.0 (reference)	1.0 (reference)	1.0 (reference)
Ever use	Any	1.23 (1.09–1.38)	1.14 (1.01–1.28)	1.48 (1.30–1.69)	1.41 (1.22–1.62)
	Low dose	1.33 (1.08–1.64)	1.24 (1.00–1.53)	1.80 (1.38–2.36)	1.72 (1.31–2.26)
	Medium dose	1.22 (1.07–1.38)	1.12 (0.98–1.28)	1.43 (1.25–1.65)	1.36 (1.18–1.58)
	High dose	0.95 (0.36–2.54)	0.86 (0.32–2.30)	1.38 (0.84–2.26)	1.29 (0.78–2.13)
	Simvastatin	1.19 (1.04–1.36)	1.10 (0.96–1.27)	1.41 (1.21–1.65)	1.36 (1.16–1.59)
	Pravastatin	1.21 (0.65–2.27)	1.14 (0.61–2.13)	0.70 (0.22–2.17)	0.66 (0.21–2.05)
	Fluvastatin	NA	NA	NA	NA
	Atorvastatin	1.31 (1.08–1.59)	1.20 (0.99–1.46)	1.53 (1.25–1.88)	1.44 (1.17–1.78)
	Rosuvastatin	1.46 (0.98–2.16)	1.33 (0.90–1.97)	2.17 (1.53–3.07)	2.04 (1.43–2.89)
Current use	Any	1.50 (1.03–2.18)	1.41 (0.97–2.05)	1.50 (1.31–1.71)	1.43 (1.24–1.65)
	0–1 years	1.42 (1.09–1.86)	1.32 (1.01–1.73)	2.13 (1.66–2.73)	2.05 (1.59–2.64)
	1–2 years	1.62 (1.21–2.17)	1.51 (1.12–2.02)	1.64 (1.21–2.22)	1.57 (1.16–2.14)
	2–3 years	1.15 (0.82–1.63)	1.06 (0.75–1.50)	1.48 (1.08–2.04)	1.39 (1.00–1.92)
	>3 years	1.28 (1.07–1.52)	1.15 (0.96–1.38)	1.30 (1.09–1.53)	1.23 (1.03–1.47)
Former use	Any	1.18 (0.87–1.60)	1.09 (0.80–1.49)	1.27 (0.88–1.83)	1.19 (0.83–1.72)
	<1 year ago	1.02 (0.59–1.77)	0.97 (0.56–1.68)	0.99 (0.53–1.86)	0.93 (0.50–1.74)
	1–2 years ago	1.44 (0.85–2.46)	1.37 (0.81–2.34)	0.84 (0.37–1.87)	0.79 (0.35–1.77)
	>2 years ago	0.99 (0.62–1.58)	0.94 (0.59–1.51)	1.29 (0.78–2.12)	1.22 (0.74–2.02)

^a^Multivariable-adjusted model adjusted for age, BMI, METs, Charlson index, pack-years of smoking, energy intake, oral corticosteroid use, quinolone antibiotic use, diabetes mellitus, renal insufficiency and education level.

### Statin use and achilles tendon pathology

**Figure 3:**
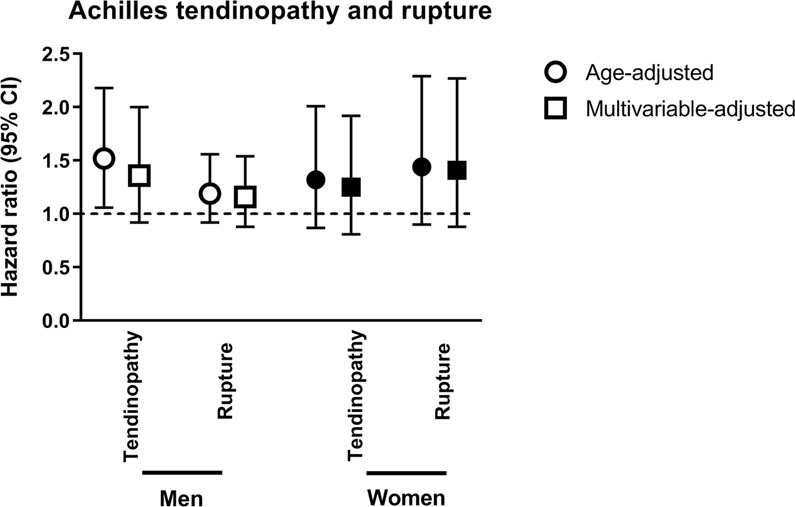
Adjusted hazard ratios (HRs) and 95% confidence intervals (CIs) for Achilles tendinopathy and Achilles tendon rupture. Adjusted hazard ratios (HRs) and 95% confidence intervals (CIs) for Achilles tendinopathy and Achilles tendon rupture in ever users of statins (both current and former users) compared with never users (hazard ration 1.0). Circles indicate age-adjusted and squares multivariable-adjusted results. The multivariable-adjusted analyses were adjusted for age, BMI, METs, Charlson index, pack-years of smoking, energy intake, oral corticosteroid use, quinolone antibiotic use, diabetes mellitus, renal insufficiency and education level. The results are presented in white figures for men and black figures for women.

In these two cohorts few of the participants had had Achilles tendinopathy or Achilles tendon rupture as a diagnosis. Tendinopathy was seen in only 88 women and 131 men and ruptures in only 71 women and 237 men, which limit the precision of our estimates. Still, there was a tendency of an increased risk for tendinopathy in men and women who had ever used statins. The aHR for men was 1.36 (95% CI: 0.92–2.00) and 1.25 (95% CI: 0.81–1.92) for women (Fig. 3). The corresponding aHR for an Achilles tendon rupture was 1.16 in men (95% CI: 0.88–1.54) and 1.41 in women (95% CI: 0.88–2.27). No further subanalyses were done because of the small number of observations.

### Simvastatin *in vitro* triggers a release of MMP-1 and MMP-13

**Figure 4:**
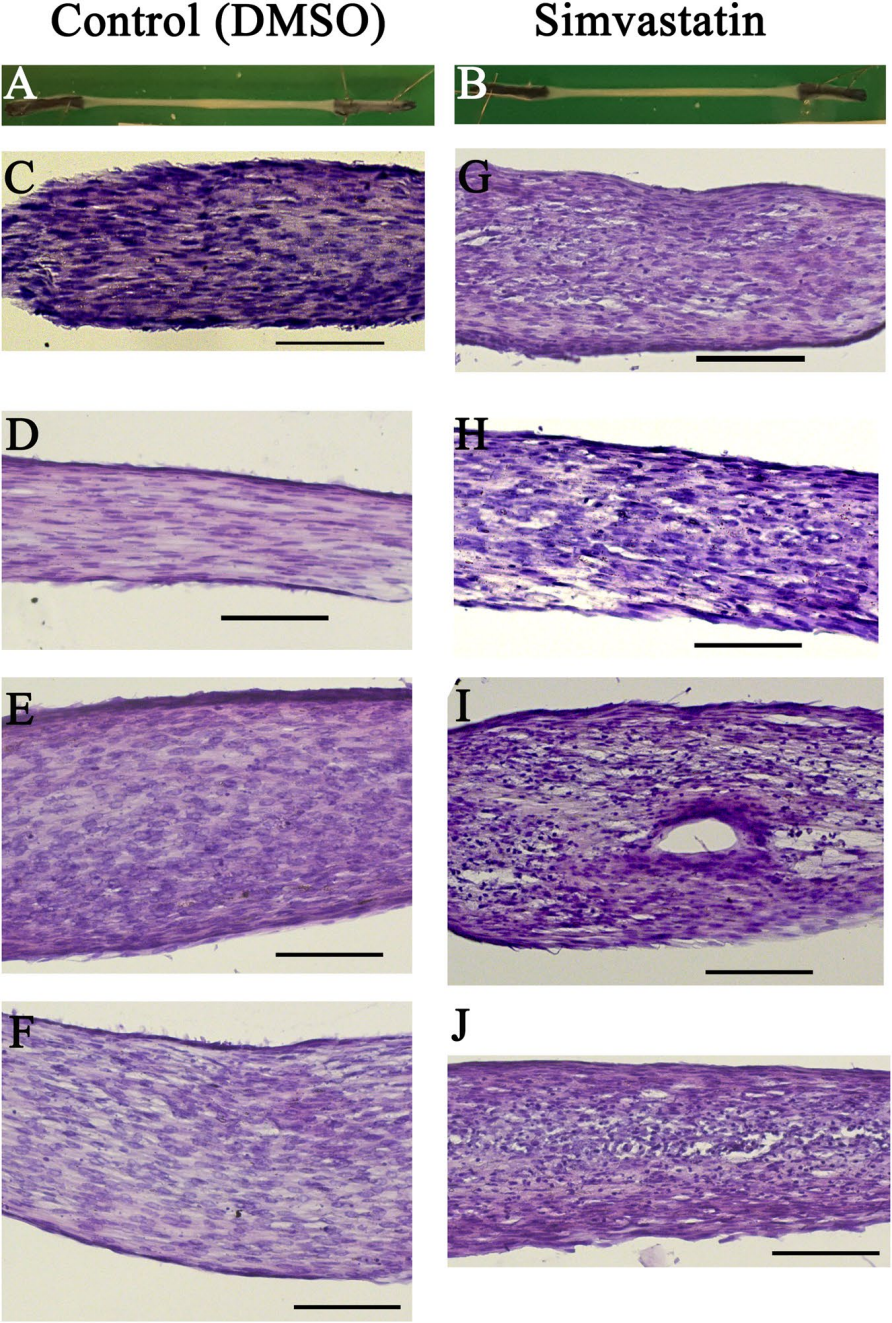
Photographs and histological images of constructs treated with simvastatin or controls. Photographs of constructs treated with DMSO (**A**) or simvastatin (**B**) for 7 days showing gross morphology. Histological images of constructs from 4 different cell donors after 7 days of DMSO (**C–F**) or simvastatin (**G–J**) exposure. 20X magnification and the line represents 100 um.

**Figure 5:**
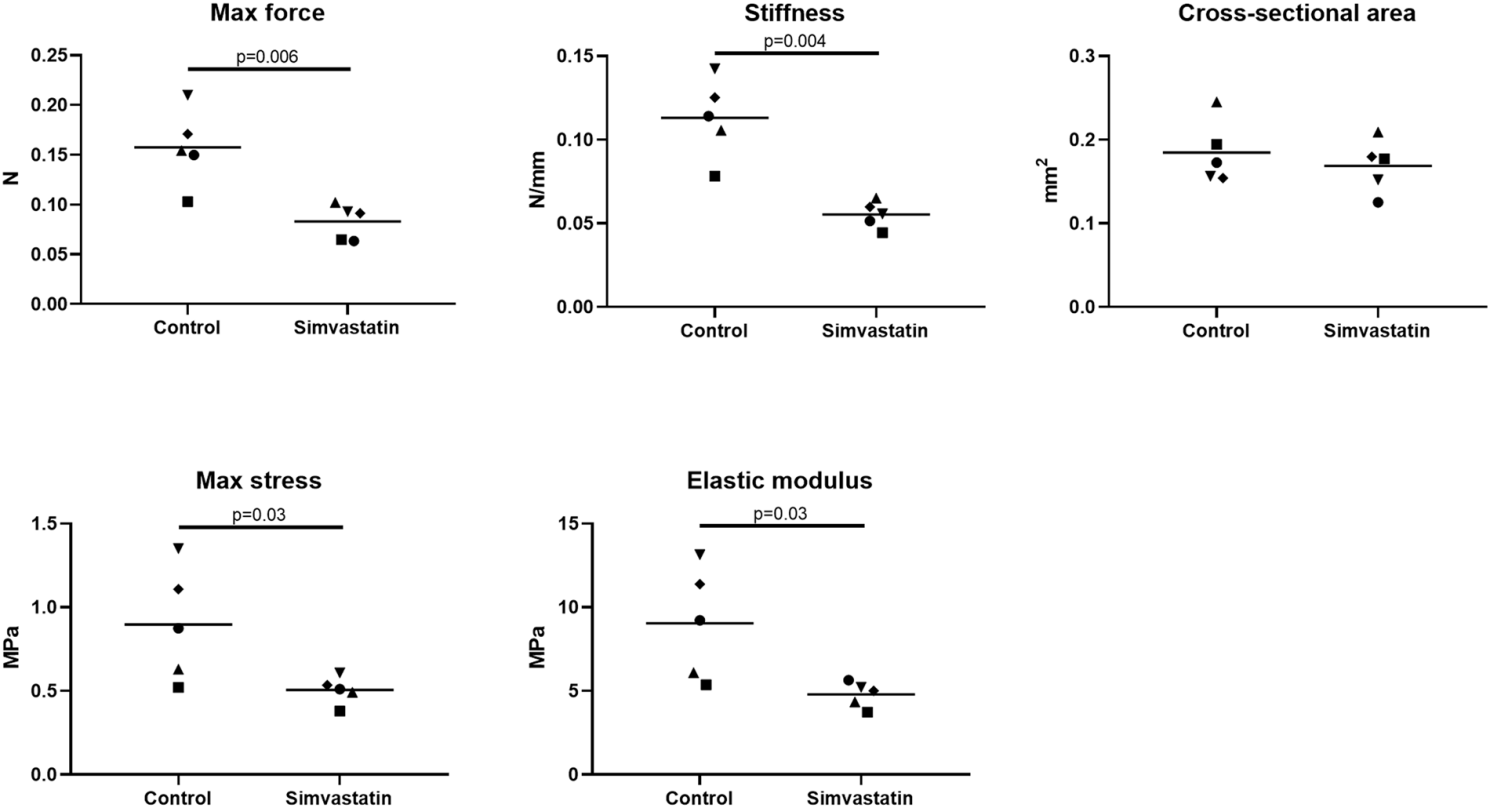
Construct mechanical data. Maximum force, maximum stiffness, cross-sectional area, maximum stress and maximum modulus of tendon constructs with or without simvastatin for 7 days. Control samples were treated with a low dose of DMSO. n = 5 different cell donors which are assigned different symbols. The line represent the mean. Data was analyzed with paired Student’s t tests (two-sided) and significance level was set at p < 0.05. The maximum force, stiffness, stress and modulus were all reduced after 7 days of simvastatin while cross-sectional  area was unaffected.

**Figure 6:**
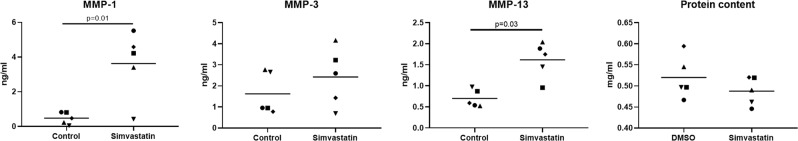
MMP and total protein release in cell culture supernatant. Levels of MMP-1, MMP-3, MMP-13 and total amount of protein released in the cell culture supernatant in samples treated with low dose DMSO (control) or simvastatin as measured by ELISA or protein quantification. n = 5 different cell donors which are assigned different symbols. The line represents the mean. Data was analyzed with paired Student’s t tests (two-sided) and significance level was set at p < 0.05. The levels of MMP-1 and MMP-13 were increased after simvastatin, whereas the levels of MMP-3 were unaffected.

We used a three-dimensional (3D) cell culture model with artificial tendons, to mechanistically investigate whether MMPs were involved in the adverse effect of statins (Fig. 4A,B). Simvastatin administration for 7 days led to a reduction in maximum force and stiffness by approximately half (both p-values < 0.005) without altering the cross-sectional area (p = 0.28, Fig. 5). The material properties, peak stress and elastic modulus were also reduced (both p-values = 0.03). Protein analyses of the cell culture supernatant showed no overall increase in protein levels but specific increase in levels of MMP-1 by a 6-fold and MMP-13 by 1.3-fold after statin treatment (both p-values < 0.03), whereas levels of MMP-3 were virtually unchanged (Fig. 6). Histological images with hematoxylin and eosin (H&E) staining confirmed a more disrupted matrix appearance after simvastatin exposure (Fig. 4C–J).

## Discussion

We found a higher risk of trigger finger and shoulder tendinopathy in current statin users but not in former users in two large population-based cohorts. Moreover, our *in vitro* studies confirmed an adverse effect of simvastatin on tendon extracellular matrix, as well as an increased release of the collagenases MMP-1 and MMP-13 by human tendon fibroblasts.

A clear, independent higher rate in the development of tendinopathy during statin use, including trigger finger, has not previously been described. However, a possible association between statin use and tendon complications has frequently been discussed^5,7,9,10,13,19^. Although tendinopathy (e.g., tendinitis) has been proposed as a more common side effect of statins than ruptures^7^, previous cohort studies have concerned only a link between statin use and tendon ruptures and observed no overall effect of statins^9,11–14^.

Hypercholesterolemia is a potential risk factor for tendinopathy^12,20–22^, and therefore it can be hard to separate the effects of hypercholesterolemia from those of statins. Nevertheless, we found an increased risk of tendinopathy only in current statin users and not former users, which supports a role of statin use in tendinopathy development. It has previously been described that most patients experience their tendon problems within the first year after statin initiation^5,7^, with a median time to onset of 8 months after initiation^7^. We believe that this finding can explain the lack of duration effect in our study. The finding also indicates that tendinopathy might be triggered in predisposed individuals, similar to what has been suggested for muscle related side-effects^3^. A previous study based on pharmacovigilance data reported a median of 23 days for tendon problem manifestation to disappear after statin cessation^7^ and a re-occurrence after statin treatment was re-introduced^7,8^.

We noted no association with a more pronounced risk after a longer treatment duration and no clear dose-response relationship. Our findings support case reports and pharmacovigilance data which have shown that 59% of the patients experienced problems within the first year of statin treatment^7,9^. A possible explanation for the weakened relation over time might be pre-disposition in certain patients to tendon-related side effects. Once these patients have been diagnosed in a closed cohort, fewer predisposed patients are at risk to develop symptoms during an expanded follow-up time. Pre-disposing factors, such as genetic susceptibility, need to be identified, as for muscle related problems^3^.

We were able to confirm a detrimental effect of simvastatin, the most commonly used statin, on artificial tendons from human tendon fibroblasts. Simvastatin is a lipophilic drug which can passively diffuse through the cell membrane into other tissues e.g. muscle and tendons^3^. Both structural and material properties of the tendons were reduced but the cross-sectional area was unaltered. A previous study using the same *in vitro* model showed no effect on collagen content after simvastatin administration but increased gene expression levels for MMP-1, MMP-3 and MMP-13^18^. Our study confirmed increased protein levels of MMP-1 and MMP-13 in the cell culture supernatant, whereas MMP-3 protein expression remained unaltered. Additionally, the total protein content in the supernatant was unaffected by simvastatin exposure. MMP-1 and MMP-13 are collagenases and the main enzymes degrading collagen type I in tendons^23^, and have previously been suggested to play a role in shoulder tendinopathy^24–26^. Although the levels of total collagen do not appear to be altered by simvastatin^18^, the integrity of the collagen could still have been affected. Our histological analysis confirmed this by showing a more disrupted appearance in the matrix after simvastatin exposure. Our results suggest that the weakened mechanical properties of tendon tissue could be caused by a disturbed matrix metalloproteinase balance. Furthermore, a weakened tendon tissue is likely more prone to injuries.

Our study design has several strengths. We used two population-based cohorts and register information for men and women rendering similar covariate information retrieved before the diagnosis of tendinopathies. Statin use and tendinopathies were identically defined in both cohorts, with complete identification of statin use and specialist physician first diagnosed cases by use of national registers (The Swedish National Patient register, the Swedish Prescribed Drug register and the Cause of death register) and the unique personal identification number provided to all Swedish residents. The Swedish Patient register has high accuracy with a positive predictive value of 97% for musculoskeletal diagnoses^27^. Moreover, we used a time to event analysis that included a time-dependent exposure analysis limiting the likelihood of a reverse causation phenomenon and immortal time bias^28,29^, a possible explanation for findings of apparent associations with different diseases by statins observed when using a cruder analytical approach^12,29^.

Our study also has some limitations. We used data from the Swedish National Patient Register, a register that only includes diagnoses ascertained by specialized physicians and not by primary health care. Therefore, a diagnosis made only by a general practitioner will be overlooked and the number of actual cases is most likely underestimated. However, this possibility is unlikely to be related to statin use. Diagnostic errors are in a similar manner probably to be found in both users and non-users. Of note, we found the most evident association between statin use and trigger finger, which has a more characteristic clinical picture than the other tendinopathies investigated. This diagnosis is therefore probably less susceptible to misclassification^30^. Additionally, due to low frequency of users, we had low to moderate precision for the estimation of associations for pravastatin, fluvastatin and rosuvastatin. Confounding by indication is another concern, where it is plausible that patients who were prescribed statins had a different risk profile than those who did not receive statins. Using our study cohort data, we were able to account for several important potential covariates known to influence tendon tissues. However, we lack information about lipid profiles in these patients and hypercholesterolemia is a possible risk factor for tendon injuries.

In conclusion, statin use appears to be associated with a higher risk of developing tendinopathy, in both men and women. We suspect that this adverse effect might be linked to an excessive MMP release followed by a weakened tendon matrix.

## Methods

### Study design

Two independent cohorts, combined with three national registers, were used to evaluate if statin use is involved in the development of tendon disorders. This was combined with an experimental study on human tendon fibroblasts, *in vitro*.

### Observational studies

The observational part is based on 92 933 participants from two population-based cohorts in Sweden, which have previously been described^31,32^: the Swedish Mammography Cohort (SMC) and the Cohort of Swedish Men (COSM) – both parts of the national research infrastructure SIMPLER (www.simpler4health.se). The study has been approved by the regional research ethical review board at the Karolinska Institutet, Stockholm (2009/1682-31; 2009/1935-32; 2013/1605-32/5; 2016/1954-32).

#### SMC

All women in Västmanland and Uppsala Counties in Sweden, born between 1914 and 1948, were invited in 1987 through 1990 to participate in the SMC^32^. Of the 90 303 women invited, 74% (n = 66 651) accepted and completed a first self-administered questionnaire on diet, alcohol consumption, education, living conditions, body weight and height. In 1997, the 56 030 women who were still alive and living in the study area received a second, expanded self-administered questionnaire. This expanded version included information on smoking status, physical activity and other lifestyle factors. In this second phase 70% (n = 39 227) of the women responded.

#### COSM

All men in Västmanland and Örebro Counties in Sweden, born between 1918 and 1952, were invited in 1997 to participate in the COSM^32^. Of the 100 303 eligible men, 49% (n = 48 850) accepted and completed a self-administered questionnaire on diet, alcohol consumption, education, living conditions, body weight and height, physical activity, smoking habits and other lifestyle factors.

### Exposure definition

Data on exposure to statins were retrieved from The Swedish Prescribed Drug Register. This register includes all prescriptions dispensed in Sweden since July 2005. All drugs in the register are classified using the Anatomical Therapeutic Chemical (ATC) system and the register is almost complete for the entire population in Sweden (patient identity data are missing for <0.3% of all items).

We defined statin exposure as any use of statins registered with ATC codes C10AA01 to C10AA08 from 1 July 1 2005 until 31 December 2014. Current users were analyzed in duration categories (<1, 1–2, 2–3, >3 years). If an individual did not collect a new prescription at the pharmacy 90 days after the previous doses would have been consumed, we regarded this individual as a former user from that point onwards. The total duration of use was calculated as the difference between the first day of dispensing and the last day of dispensing plus 3 months, excluding gaps in treatment. The drugs are normally dispensed every third month. The individual statins that were analyzed were pravastatin and rosuvastatin (both hydrophilic) and simvastatin, fluvastatin and atorvastatin (all lipophilic statins). We additionally classified statin dose per day into three categories: low dose (simvastatin 5–10 mg, pravastatin 10 mg, atorvastatin 10 mg, rosuvastatin 5–10 mg), medium dose (simvastatin 20–40 mg, pravastatin 20–40 mg, fluvastatin 20–40 mg, atorvastatin 20–40 mg, rosuvastatin 20 mg) and high dose (simvastatin 80 mg, fluvastatin 80 mg, atorvastatin 80 mg, rosuvastatin 40 mg).

### Outcome definition

We defined the outcome as the first occurrence of any of our diagnosis in inpatient or outpatient specialist care data after the index date 1 July 2005 (starting the first day of the prescription register) until 31 December 2014. First occurrence of trigger finger, tendinopathy or Achilles tendon rupture was considered an outcome based on ICD-10 codes: M65.3 for trigger finger, M75.1, M75.2, M75.3, M75.4 and M75.5 for shoulder tendinopathy, M76.6, including sub-diagnosis under that coding for Achilles tendinopathy and S86.0, M66.3 H and M66.2 H for Achilles tendon rupture. Those patients with a tendinopathy diagnosed before the index date were excluded from the analysis.

### *In vitro* studies with tendon constructs

We isolated tendon fibroblast by collagenase digestion from small segments of human semitendinosus tendons in five patients undergoing anterior cruciate ligament reconstructive surgery as previously described^31^. The experiments were approved by the regional ethics review board in Linköping, Sweden (2015/408-31) and patients gave written informed consent to participation. The investigation has been conducted according to the Declaration of Helsinki. Cells were seeded in flasks and cultured to confluence in DMEM/F12 supplemented with 10% fetal bovine serum (FBS) and 1% Penicillin-Streptomycin.

### Tendon construct formation

Tendon constructs were assembled as previously described with small modifications^18^. The bottom of six-well plates were coated with silicon (SYLGARD, Dow-Chemicals) and two silk sutures (0.5 cm, Ethicon) were pinned onto this as anchor points (15 mm apart). Then, 250 000 cells (passage 2–5) were mixed into a fibrin gel and quickly spread in each well. The fibrin was left to set before it was covered with DMEM/F12 (supplemented with 10% FBS, 0.2 mM L-ascorbic acid 2-phosphate, 0.05 mM L-Proline and 1% Penicillin-Streptomycin). The cell culture supernatant was replaced every second to third day and adhesions to the side of the well were detached 2 days after seeding using a fine pipette tip to allow gel contraction. After 8–14 days, the cells had contracted the structure to a rod-like shape in between the anchors. After 14 days, statin treatment was introduced. Constructs were treated with either 0.5 µM of simvastatin which was dissolved in DMSO (Sigma Aldrich, S6196) for 7 days or 0.02% of DMSO (controls) and the serum levels were reduced to 5%.

### Mechanical testing of the constructs

Tensile testing was performed in an Instron 3343 Single Column Testing System (10 N load cell) with a BioPuls^TM^ liquid chamber and Bluehill 2^®^ material testing software (version 2.35.917, Instron, Buckinghamshire, United Kingdom). Construct diameter and length were measured before testing by capturing images with a digital camera at 11 cm from the construct. The diameter was measured in four places (the thickest and thinnest places on the right and left side of the construct) using ImageJ (NIH, USA). An average cross-sectional area was calculated assuming a circular cross section. Each picture also included a reference with known diameter and length. The sutures, in each end of the construct, were glued between sandpaper sheets to ensure adhesion within the clamp. The specimen was then transferred to a bath containing PBS at 30 °C, and after a short adaptation period, the test was started. The samples were stretched at 4 mm/min until failure. The testing was performed in quadruplicate for each cell donor treated with simvastatin and in duplicate for each cell donor treated with DMSO.

### MMP ELISA and total protein content measurement

Cell culture supernatant was stored at −70 °C until analysis. Levels of the collagenases (MMP-1 and MMP-13) and stromelysin 1 (MMP-3) were determined using sandwich ELISA kits (Catalogue #DMP100, DM1300, DMP300; R&D Systems) according to the manufacturer’s instructions. Total protein content in the supernatant was measure by the Bio-Rad protein assay according to the manufacturer’s instructions (Bio-Rad).

### Histology

Constructs were fixed in 4% paraformaldehyde, followed by dehydration in a series of increasing concentrations of ethanol, before they were embedded in paraffin. The paraffin blocks were then sectioned longitudinally, in 7 µm sections and stained with routine hematoxylin and eosin. The sections were observed in a conventional light microscope.

### Statistical analysis

#### Observational study

All cohort participants who were alive and resident in Sweden at the starting point (1 July 2005) were included in the analysis. We estimated age- and multivariable-adjusted hazard ratios (HRs) for relative risk of trigger finger, shoulder tendinopathy, Achilles tendinopathy and Achilles rupture in users versus non-users of statins using Cox proportional hazards regression analysis. We used years of observation as a time scale and with time-updated information on exposures. All individuals were followed from 1 July 2005 until date of outcome, date of death, emigration, or to the end of the observation period (i.e. 31 December 2014), whichever occurred first.

To minimize potential confounding a multivariable model ‒ identified by a directed acyclic graph approach^33^ ‒ was used that included the following variables: age (continuous), body mass index (weight [kg] divided by the height [m] squared, continuous), validated total physical activity (continuous as metabolic equivalent-hours/day)^34–36^, educational level (≤9 years, 10–12 years, >12 years and other education such as vocational) as a marker of socioeconomic status, smoking status categorized as never, former or current smoking, pack-years of smoking (continuous), total energy intake as an additional proxy variable for physical activity^37^ (continuous), weighted Charlson comorbidity index (continuous)^38,39^, corticosteroid use (ever/never), quinolone use (ever/never), diabetes mellitus (yes/no by self-report or by ICD-10 codes E10-E14 identified from the National Patient Register) and renal insufficiency (yes/no by ICD-10 codes N17-N19 identified from the National Patient Register). Analyses were performed with SAS 9.4. Lifestyle information was collected from the latest questionnaire cycle (i.e. 1997).

#### *In vitro* study

Results from mechanical testing, ELISA and protein content were analyzed with paired Student’s t tests (two-sided). The significance level was set at p < 0.05.

## Data Availability

Data cannot be made freely available as they are subject to secrecy in accordance with the Swedish Public Access to Information and Secrecy Act, but can be made available to researchers upon request (after subject to a review of secrecy and ethical approval) by contact to the national research infrastructure SIMPLER (Swedish Infrastructure for Medical Population-based Life-course and Environmental Research): www.simpler4health.se.
